# Identification and characterization of latency-associated peptide-expressing γδ T cells

**DOI:** 10.1038/ncomms9726

**Published:** 2015-12-08

**Authors:** Rafael M. Rezende, Andre P. da Cunha, Chantal Kuhn, Stephen Rubino, Hanane M’Hamdi, Galina Gabriely, Tyler Vandeventer, Shirong Liu, Ron Cialic, Natalia Pinheiro-Rosa, Rafael P. Oliveira, Jellert T. Gaublomme, Nikolaus Obholzer, James Kozubek, Nathalie Pochet, Ana M. C. Faria, Howard L. Weiner

**Affiliations:** 1 Ann Romney Center for Neurologic Diseases, Brigham and Women’s Hospital, Harvard Medical School, Boston, Massachusetts 02115, USA; 2 Rheumatology Unit, Department of Medicine at Karolinska University Hospital, Karolinska Institute, Solna, Stockholm 17177, Sweden; 3 Department of Biochemistry and Immunology, Institute of Biological Sciences, Federal University of Minas Gerais, Belo Horizonte 31.270-901, Brazil; 4 Department of Chemistry and Chemical Biology, Harvard University, Cambridge, Massachusetts 02138, USA; 5 Department of Physics, Harvard University, Cambridge, Massachusetts 02138, USA; 6 Program in Translational NeuroPsychiatric Genomics, Institute for the Neurosciences, Departments of Neurology and Psychiatry, Brigham and Women’s Hospital, Boston, Massachusetts 02115, USA; 7 Broad Institute of Massachusetts Institute of Technology (MIT) and Harvard, Cambridge, Massachusetts 02142, USA

## Abstract

γδ T cells are a subset of lymphocytes specialized in protecting the host against pathogens and tumours. Here we describe a subset of regulatory γδ T cells that express the latency-associated peptide (LAP), a membrane-bound TGF-β1. Thymic CD27+IFN-γ+CCR9+α_4_β_7_+TCRγδ+ cells migrate to the periphery, particularly to Peyer’s patches and small intestine lamina propria, where they upregulate LAP, downregulate IFN-γ via ATF-3 expression and acquire a regulatory phenotype. TCRγδ+LAP+ cells express antigen presentation molecules and function as antigen presenting cells that induce CD4+Foxp3+ regulatory T cells, although TCRγδ+LAP+ cells do not themselves express Foxp3. Identification of TCRγδ+LAP+ regulatory cells provides an avenue for understanding immune regulation and biologic processes linked to intestinal function and disease.

## Abstract (web summary)

Latency-associated peptide (LAP) is a membrane-bound form of TGF-β1. Here the authors show that LAP marks a subset of regulatory γδ T cells with innate gut-homing properties, which present antigen and induce CD4+ Foxp3+ in Peyer's patches and lamina propria.

## Graphical abstract

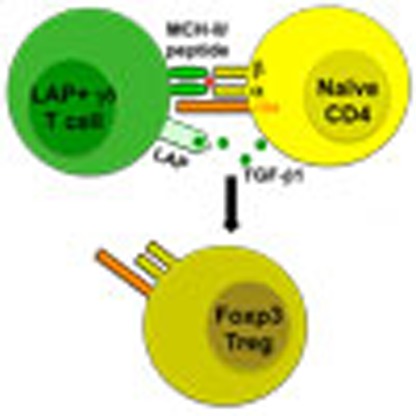

Gamma-delta (γδ) T cells are lymphocytes bearing a T-cell receptor composed of gamma and delta chains as opposed to alpha and beta chains found in conventional CD4+/CD8+ T cells. Despite comprising the majority of immune cells in niches associated with epithelial surfaces such as the intestine, only 1–2% of γδ T cells are present in secondary lymphoid tissues^1^. γδ T cells are considered the first line of defense against pathogens as they can rapidly respond to TCR signals in an MHC-independent manner^2^ and to pattern recognition receptor signals such as Toll-like receptors^3^. Upon activation, γδ T cells rapidly secrete IFN-γ and IL-17 and acquire cytotoxic activity^4^,^5^,^6^. Two distinct γδ T cell subsets have been described on the basis of their cytokine production profile. γδT1 cells express CD27 and secrete IFN-γ (ref. 7), whereas γδT17 cells are CD27−, express CCR6 and secrete IL-17 (ref. 6).

In addition to their physiologic functions, γδ T cells may participate in immunopathology, including autoimmune disease models such as experimental autoimmune encephalomyelitis (EAE)^8^ and arthritis^9^. As γδ T cells are particularly abundant in the intestinal mucosa, their participation in intestinal inflammation has also been described^10^,^11^. IL-17+ γδ T cells play a crucial role in enhancing *in vivo* Th1 and Th17 differentiation and T cell-mediated colitis in mice^10^ and exacerbate intestinal inflammation induced by dysregulated immune homeostasis^11^.

γδ T cells have also been reported to have immunoregulatory function. For example, in inflammatory bowel disease models, γδ T-cell-deficient mice develop spontaneous colitis and are susceptible to 2,4,6-trinitrobenzene sulfonic acid-induced colitis^12^. Transfer of intraepithelial γδ lymphocytes (IEL-γδ) ameliorates colitis in this model^12^. In dextran sodium sulfate (DSS)-induced colitis in mice, IEL-γδ T cells help preserve the integrity of damaged epithelial surfaces by the localized delivery of keratinocyte growth factor, a potent intestinal epithelial cell mitogen^13^. Furthermore, by secreting IL-22 as well as anti-microbial products in a retinoic acid-dependent fashion, γδ T cells play an important role in the attenuation of intestinal inflammation induced by DSS or *Citrobacter rodentium* infection in mice^14^. Oral tolerance, a physiologic process that helps maintain gut homeostasis to the daily challenge of microbiota and dietary antigens^15^ is impaired in mice depleted of γδ T cells or in γδ T-cell-deficient mice^16^,^17^.

The mechanism(s) by which γδ T cells exert regulatory function is not well understood. Forkhead box p3 (Foxp3) expression is not observed in murine γδ T cells *ex vivo* though they may express Foxp3 *in vitro* when cultured in the presence of TGF-β1 (ref. 18). There are low levels of Foxp3 expression in human γδ T cells that, like in mice, increase under Treg-inducing conditions *in vitro*^18^,^19^. Moreover, Rhodes *et al.*^20^ reported the existence of an IL-10+ γδ T cell subset that protected the liver from Listeria-induced, CD8+ T-cell-mediated injury in mice. Interestingly, activated γδ T cells from cattle^21^, mice^22^ and humans^23^ have been shown to express high levels of MHC-II and co-stimulatory molecules and function as antigen presenting cells (APCs). Although this APC-like function of γδ T cells has been associated with a more pro-inflammatory immune response^24^, it is possible that regulatory subtypes of γδ T cells may occur *in vivo* and have immunoregulatory function.

In the present study, we describe and characterize a subset of regulatory γδ T cells that are Foxp3 negative and express membrane-bound TGF-β1 in the form of latency-associated peptide (LAP). These cells function as APCs and possess the ability to induce Foxp3 in CD4 T cells *in vitro* and *in vivo*.

## Results

### Identification of a subset of LAP-expressing γδ T cells

**Figure 1:**
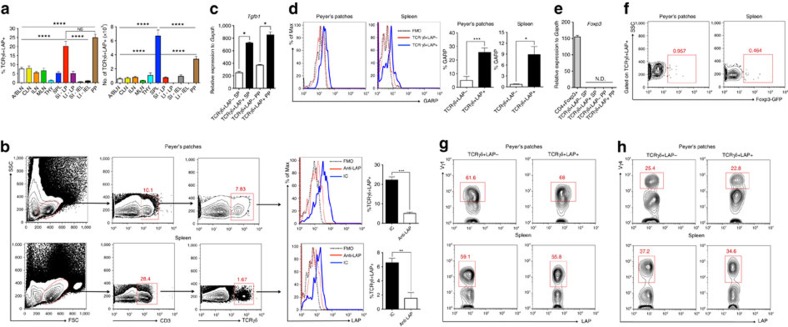
γδ T cells express the latency-associated peptide (LAP), but not Foxp3. (**a**) Frequency and absolute number of γδ T cells expressing LAP (CD3+TCRγδ+) from axillary/brachial (A/BLN), cervical (CLN), inguinal (ILN), mesenteric (MLN) lymph nodes, thymus (THY), spleen (SPL), small (SI-LP) and large intestine (LI-LP) lamina propria, small (SI-IEL) and large intestine (LI-IEL) intraepithelial lymphocytes and Peyer’s patches (PPs) from naive C57BL/6 mice (*n*=15). These experiments were performed at least 20 times. (**b**) LAP-gating scheme in PPs and spleen. Cells were first incubated with an unconjugated anti-LAP antibody (clone TW7–16B4 or isotype control (IC) antibody) for 20 min to block LAP and then a conjugated anti-LAP antibody (same clone, TW7–16B4) was added to establish the specificity of LAP staining on γδ T cells. (**c**) Quantitative RT-PCR analysis of *Tgfb* mRNAs from TCRγδ+LAP− and TCRγδ+LAP+ cells (PP and spleen) of naive C57BL/6 mice (*n*=pooled cells from 10 mice per experiment). These data are representative of at least 3 independent experiments. (**d**) GARP expression on CD3+TCRγδ+LAP− and CD3+TCRγδ+LAP+ cells from PP and spleen of naive C57BL/6 mice (*n*=6). These data are representative of at least 3 independent experiments. (**e**) Quantitative RT–PCR analysis of *Foxp3* mRNAs from CD3+TCRγδ+LAP−, CD3+TCRγδ+LAP+ cells (PPs and spleen) and CD4+Foxp3+ cells (Foxp3 positive control) of naive Foxp3-GFP mice (*n*=pooled cells from 10 mice per experiment). These data are representative of at least five independent experiments. (**f**) Foxp3 expression in γδ T cells (CD3+TCRγδ+LAP+) from PPs and spleen of naive Foxp3-GFP mice (*n*=6). These data are representative of at least five independent experiments. (**g**,**h**) Vγ1 (**g**) and Vγ4 (**h**) TCR chains expression on CD3+TCRγδ+LAP+ cells from PP and spleen of naive C57BL/6 mice (*n*=6). These data are representative of at least three independent experiments. Data are shown as mean±s.e.m. One-way analysis of variance (**a**) and Student’s *t*-test (**b**,**d**) were used. *P<0.05, **P<0.01, ***P<0.001, ****P<0.0001.

Given our interest in the regulatory function of T cells, which express membrane-bound TGF-β1 through its accessory-binding molecule LAP^25^,^26^, we investigated LAP expression on γδ T cells. We found LAP-expressing γδ T cells (TCRγδ+LAP+) in several mouse lymphoid organs, particularly those from Peyer’s patches (PPs) and small intestine lamina propria (SI-LP), where ∼20% of the γδ T cells were positive for this molecule (Fig. 1a,b; Supplementary Fig. 1a). Owing to the high cellularity in spleen, the absolute number of TCRγδ+LAP+ cells in the spleen was greater than in the other organs investigated, followed by PPs (Fig. 1a). The absolute number of TCRγδ+LAP+ cells in SI-LP was as low as in lymph nodes, thymus, large intestine lamina propria (LI-LP) and intraepithelial lymphocytes (Fig. 1a). There was minimal expression of LAP on γδ T cells from thymus, intraepithelial lymphocytes and LI-LP (Fig. 1a; Supplementary Fig. 1b). LAP was less expressed on other lymphoid cells in the PPs and SI-LP, such as CD4 and CD8 T cells, as compared with LAP expression on γδ T cells from these same organs (Fig. 1a; Supplementary Fig. 2a,b). We also detected LAP on γδ T cells from human peripheral blood (Supplementary Fig. 3a,b), at comparable percentages found on splenic γδ T cells from mice. Because the highest frequency and absolute number of TCRγδ+LAP+ cells were found in PPs and spleen, respectively, we performed our characterization using TCRγδ+LAP+ cells from these organs. Consistent with their LAP expression, mouse TCRγδ+LAP+ cells expressed more TGF-β1 than TCRγδ+LAP− cells, as measured by messenger RNA (mRNA; Fig. 1c), and also expressed the cell-surface molecule Glycoprotein A Repetitions Predominant (GARP), which is known to bind and attach LAP to the cell surface^27^ (Fig. 1d). It has been reported that under Foxp3-induction conditions *in vitro*, both human and mice γδ T cells express Foxp3 (refs 18, 19). However, Foxp3 was not detected *ex vivo* in non-manipulated naive mice^18^. Consistent with this, we found that γδ T cells from PPs and spleen of naive Foxp3-GFP mice did not express Foxp3 as measured either by mRNA or protein expression (Fig. 1e,f). Vγ1 and Vγ4 TCR chains were expressed on TCRγδ+LAP+ and TCRγδ+LAP− cells, with Vγ1 the most expressed in both cell populations (Fig. 1g,h; Supplementary Fig. 4; nomenclature based on Heilig and Tonegawa^28^). In summary, our results identify a subpopulation of γδ T cells in mice that express LAP on their surface.

### TCRγδ+LAP+ cells induce Tregs and ameliorate colitis

**Figure 2:**
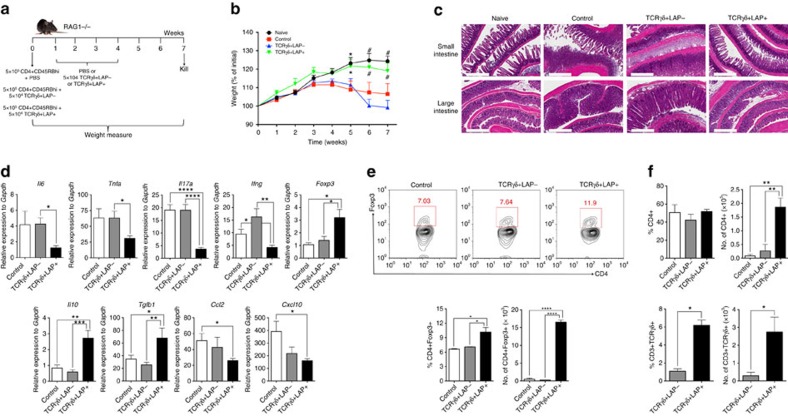
TCRγδ+LAP+ cells prevent CD4+CD45RBhigh cell transfer-induced colitis in mice. (**a**) Schematic protocol of CD4+CD45RBhigh cell transfer-induced colitis and γδ T cell adoptive transfers. (**b**) Body weight (% of initial weight) was measured throughout the experiment. Graph shows the mean±s.e.m. of naive, CD4+CD45RBhigh cell-treated only (Control) or together with CD3+TCRγδ+LAP− or CD3+TCRγδ+LAP+ cells groups. (**c**) Colons and small intestines were removed at week 7, and 5-μm serial sections were stained with haematoxylin-eosin. Magnification of × 40. Scale bars, 600 μm. (**d**) Quantitative RT–PCR analysis of pro-inflammatory and anti-inflammatory cytokine mRNAs from SI-LP of cell transfer-induced colitis mice. These data are representative of three independent experiments. (**e**,**f**) FACS plots, frequency and absolute number of Foxp3 expression in transferred CD4 T cells (**e**) and frequency and absolute number of total transferred CD4 T cells as well as total transferred CD3+TCRγδ+ cells (**f**) in SI-LP of cell transfer-induced colitis mice. These data are representative of three independent experiments. Data are shown as mean±s.e.m. (*n*=9 for naive; *n*=15 for control and TCRγδ+LAP− groups; *n*=9 for TCRγδ+LAP+ group). Two-way analysis of variance (ANOVA) (**b**) and one-way ANOVA followed by Tukey multiple comparisons (**d**–**f**) were used. *, statistically different from control group; #, statistically different from both control and TCRγδ+LAP− groups (*P*<0.05). *P<0.05, **P<0.01, ***P<0.001, ****P<0.0001.

As LAP expression confers regulatory function to CD4 and CD8 T cells^25^,^26^, we asked whether TCRγδ+LAP+ cells had *in vivo* regulatory activity. Two models of colitis were used to address this question: the T-cell model of colitis induced by CD4+CD45RBhigh cell transfer into immunodeficient mice^29^ (Fig. 2a); and the innate immune-mediated model of colitis induced by oral administration of the chemical compound DSS^30^ (Supplementary Fig. 6a). RAG-1−/− mice transferred with either CD4+CD45RBhigh cells alone (control group) or together with TCRγδ+LAP− cells began to exhibit signs of colitis as measured by body weight loss at 5 weeks after transfer. This was not observed when animals received TCRγδ+LAP+ cells (Fig. 2b). The experiment was terminated at 7 weeks at which time mice had lost 15% of their body weight. Consistent with the weight reduction, histological analyses showed more severe colonic and small intestine tissue ulceration and inflammatory cell infiltrate in control or TCRγδ+LAP− mice than TCRγδ+LAP+ cell-treated mice (Fig. 2c). Furthermore, mice transferred with TCRγδ+LAP+ cells had less IL-6, TNF-α, IL-17A and IFN-γ as well as CCL2 and CXCL10 (chemokines involved in the recruitment of myeloid and lymphoid cells to inflammatory sites) mRNA expression. Transfer of TCRγδ+LAP+ cells increased IL-10 and TGF-β1 mRNA in the SI-LP as compared with control or TCRγδ+LAP− cell-transferred animals (Fig. 2d). In the LI-LP, expression of IFN-γ mRNA was decreased in TCRγδ+LAP+ cell-treated mice compared with the other two groups, though IL-17A, CCL2 and CXCL10 mRNA levels were reduced in TCRγδ+LAP+ cell-treated animals as compared with TCRγδ+LAP− cell-transferred mice, but were not different from the control group (Supplementary Fig. 5a). Foxp3 mRNA was upregulated in the SI-LP, but not in the LI-LP of TCRγδ+LAP+ cell-treated mice (Fig. 2d; Supplementary Fig. 5a). Consistent with this, the frequency and absolute number of CD4+Foxp3+ cells in the SI-LP were higher in mice treated with TCRγδ+LAP+ cells (Fig. 2e). PP could not be investigated because RAG-1−/− mice do not develop PP. Fluorescence-activated cell sorting (FACS) analysis demonstrated that the absolute number of total CD4 T cells and the frequency/absolute number of TCRγδ+LAP+ cells were elevated compared with the LAP− counterpart in SI-LP, but not in LI-LP (Fig. 2f; Supplementary Fig. 5b). No differences were observed in either percentage or absolute number of total CD4+ and γδ T cells in the spleen (Supplementary Fig. 5c), though a significant increase of CD4+Foxp3+ cells in TCRγδ+LAP+ cell-treated mice was observed (Supplementary Fig. 5d).

We also investigated TCRγδ+LAP+ cells in the DSS-model, which is a T cell-independent model of colitis. Transfer of TCRγδ+LAP+ cells ameliorated disease as measured by body weight with initial effects observed at day 6 and more prominent effects beginning at day 10 (Supplementary Fig. 6b). Thus there appears to be a combined effect on both disease progression and recovery. In addition, colonic length was not reduced in TCRγδ+LAP+ cell-treated mice (Supplementary Fig. 6c) and histological analysis showed less tissue ulceration and inflammatory cell infiltrate in mice transferred with TCRγδ+LAP+ cells (Supplementary Fig. 6d). IFN-γ mRNA was increased in LI-LP from TCRγδ+LAP+ cell-treated mice (Supplementary Fig. 6e). These analyses were performed at day 14 after DSS treatment, which corresponds to the recovery phase of the colitis. Consistent with this, LI-LP from mice transferred TCRγδ+LAP+ cells showed higher levels of IL-10 and TGF-β1 mRNA, cytokines important for gut homeostasis, as well as IL-22, an interleukin involved in the protection of barrier surfaces, such as the gut epithelium^31^ (Supplementary Fig. 6e). FACS analyses demonstrated that the absolute number of total CD4 T cells and frequency/absolute number of CD4+Foxp3+ cells were increased in the spleen of mice treated with TCRγδ+LAP+ cells as compared with the other groups (Supplementary Fig. 6f). In PP, the absolute number of total CD4 and CD4+Foxp3+ cells was increased in TCRγδ+LAP+ cell-treated mice as compared with mice that received TCRγδ+LAP− cells, but was not different from naive or control groups (Supplementary Fig. 6g). In the LI-LP, TCRγδ+LAP− and TCRγδ+LAP+ cell-treated mice had increased frequency and absolute number of total CD4 and CD4+Foxp3+ cells, but were not different from each other (Supplementary Fig. 6h).

To investigate whether TCRγδ+LAP+ cells had suppressive properties *in vitro*, we sorted naive CD4+ T cells from Foxp3-GFP mice and stimulated them with anti-CD3ε in the presence of TCRγδ+LAP− or TCRγδ+LAP+ cells from WT mice plus antigen presenting cells (APCs). As a control, nTregs were tested. We found that neither TCRγδ+LAP+ nor TCRγδ+LAP− cells were suppressive *in vitro* as compared with nTreg cells. Instead, TCRγδ+LAP+ cells induced higher proliferation than control or TCRγδ+LAP− cells even at a 1:8 responder:TCRγδ+LAP+ cell ratio (Supplementary Fig. 7a,b). Furthermore, no Foxp3 induction in responder cells was observed when either TCRγδ+LAP+ or TCRγδ+LAP− cells were added to the culture as compared with the Foxp3 induction by nTreg cells (Supplementary Fig. 7c,d). Consistent with this, TCRγδ+LAP+ cells stimulated *in vitro* with plate-bound anti-CD3ε and anti-CD28 acquired a pro-inflammatory profile with less TGF-β1 and more TNF-α mRNA expression (Supplementary Fig. 8a,b). To further investigate whether TCRγδ+LAP+ cells had suppressive properties *in vitro* and to determine whether the activation of TCRγδ+LAP+ cells by anti-CD3ε was associated with their inability to induce CD4+Foxp3+ cells *in vitro*, we sorted naive CD4 T cells from 2D2xFoxp3-GFP mice (2D2 are MOG_35–55_-specific TCR transgenic animals) and stimulated them with MOG_35–55_ peptide in the presence of TCRγδ+LAP− or TCRγδ+LAP+ cells from wild-type (WT) mice in the absence of APCs. This allowed us to stimulate CD4+ T cells with MOG peptide and to assess the APC function of TCRγδ+LAP+ without stimulating γδ T cells with anti-CD3ε as we did above. We found that TCRγδ+LAP+ but not TCRγδ+LAP− cells induced both proliferation and Foxp3 expression in CD4 T cells (Supplementary Fig. 8c,d). Thus, when TCRγδ+LAP+ cells are not stimulated by anti-CD3ε *in vitro*, they are able to induce CD4+Foxp3+ Treg cells as they do *in vivo*. Moreover, because we did not add APCs to the co-culture, these results suggest that TCRγδ+LAP+ cells functioned as APCs and provided co-stimulatory signals to the naive CD4+ T cells (Supplementary Fig. 8c,d). In summary, we found that *in vivo* TCRγδ+LAP+ cells ameliorate colitis by promoting the induction of Foxp3 Treg cells. *In vitro* experiments demonstrate that they do not have direct regulatory function, but indirectly induce Tregs through their APC properties.

### Antigen presenting cell function of TCRγδ+LAP+ cells

**Figure 3:**
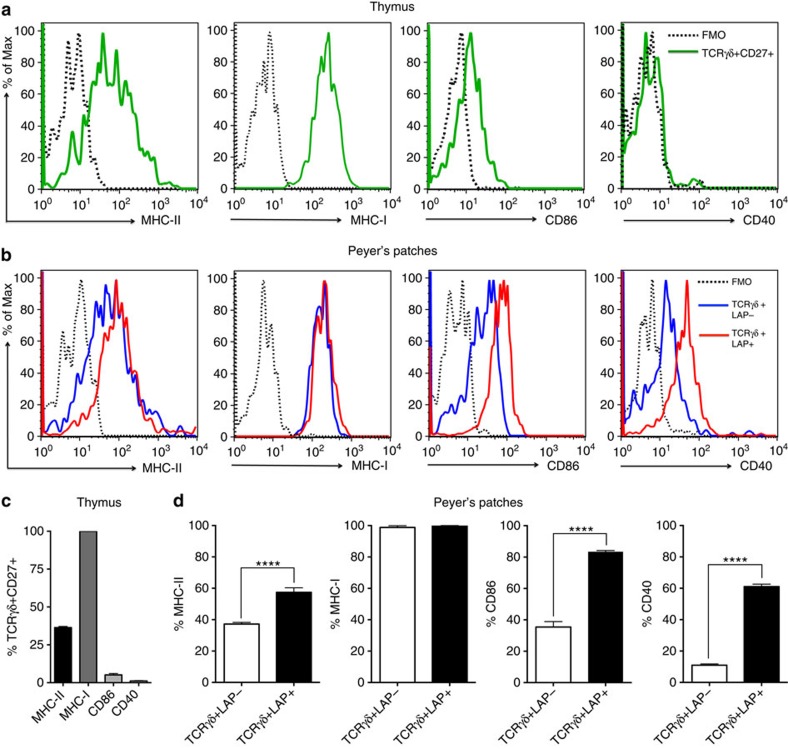
Expression of antigen presentation-related molecules in γδ T cells. (**a**,**b**) MHC-II, MHC-I, CD86 and CD40 expression on γδ T cells from thymus (**a**; CD3+TCRγδ+CD27+) and PPs (**b**; CD3+TCRγδ+LAP+) (*n*=9). (**c**,**d**) Frequency of MHC-II, MHC-I, CD86 and CD40 on γδ T cells from thymus (**c**) and PPs (**d**). These data are representative of at least five independent experiments. Student’s *t*-test was used. ****P<0.0001.

To further characterize TCRγδ+LAP+ cells, we performed RNA-Seq of both TCRγδ+LAP+ and TCRγδ+LAP− cells (Table 1; Supplementary Data 1 and Supplementary Data 2). We identified a signature of 407 genes that were enriched in TCRγδ+LAP+ versus TCRγδ+LAP− cells with *P*<0.05. Among the upregulated genes, we found increased expression of genes related to antigen presentation, including MHC class II molecules (H2-Aa, H2-Ab1, H2-Eb1 and H2-Eb2), CD40 and CD86. We confirmed the expression of these APC-associated molecules on TCRγδ+LAP+ cells by flow cytometry (Fig. 3). Thymic γδT1 cells and TCRγδ+LAP− cells from PPs expressed MHC-II, to a lesser extent CD86, but did not express CD40 (Fig. 3a–d). TCRγδ+LAP+ cells from PPs had higher expression of MHC-II and CD86 than both thymic γδT1 cells and TCRγδ+LAP− cells. They also expressed CD40 (Fig. 3a–d). MHC-I was detected on all γδ T cells analysed (Fig. 3a–d).

**Figure 4:**
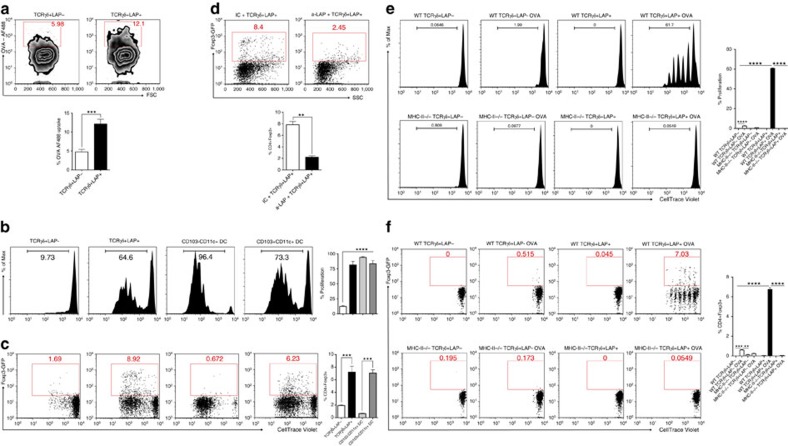
TCRγδ+LAP+ cells function as APCs and induce Foxp3 in CD4 T cells. (**a**) Soluble ovalbumin (OVA) coupled to Alexa Fluor 488 (OVA-AF488) endocytosis by CD3+TCRγδ+LAP− or CD3+TCRγδ+LAP+ cells after 3 h of culture *in vitro* at 37 °C (*n*=pooled cells from 10 mice per experiment). (**b**,**c**) Proliferation (**b**) and Foxp3 induction (**c**) in CellTrace Violet-stained naive CD4 T cells from OT-IIxFoxp3-GFP mice co-cultured with OVA_323–339_-loaded CD3+TCRγδ+LAP−, CD3+TCRγδ+LAP+, CD103−CD11c+. or CD103+CD11c+ cells from WT C57BL/6 mice for 4 days at 37 °C (*n*=pooled cells from 10 mice per experiment). (**d**) Foxp3 induction in CellTrace Violet-stained naive CD4 T cells from OT-IIxFoxp3-GFP mice co-cultured with OVA_323–339_-loaded TCRγδ+LAP+ cells from WT C57BL/6 mice in the presence or absence of 30 μg ml^−1^ of anti-LAP mAb for 4 days at 37 °C (*n*=pooled cells from 10 mice per experiment). (**e**,**f**) Proliferation (**e**) and Foxp3 induction (**f**) in CellTrace Violet-stained naive CD4 T cells from OT-IIxFoxp3-GFP mice co-cultured with OVA_323–339_-loaded (or not) CD3+TCRγδ+LAP− or CD3+TCRγδ+LAP+ cells from either WT C57BL/6 or MHC-II−/− mice for 4 days at 37 °C (*n*=pooled cells from 10 mice per experiment). These data are representative of at least three independent experiments. One-way analysis of variance followed by Tukey multiple comparisons (**b**,**e**,**f**) and Student’s *t*-test (**a**,**c**,**d**) were used. **P<0.01, ***P<0.001, ****P<0.0001.

To determine whether TCRγδ+LAP+ cells could function as antigen presenting cells, we cultured TCRγδ+LAP+ or TCRγδ+LAP− cells with Alexa Fluor 488-conjugated ovalbumin (OVA). TCRγδ+LAP+ cells took up twice as much OVA as their LAP− counterparts (Fig. 4a). When OVA_323–339_ peptide-pulsed TCRγδ+LAP+ were cultured with naive CD4+ T cells from OT-IIxFoxp3-GFP (OVA_323–339_-specific TCR transgenic) mice, we observed proliferation to a similar extent as with OVA_323–339_ peptide-pulsed CD103+CD11c+ dendritic cells (DC; Fig. 4b). No proliferation was observed when TCRγδ+LAP− cells were pulsed with OVA_323–339_ peptide and were cultured with CD4+ T cells (Fig. 4b).

The decreased proliferative response seen when T cells were cultured with CD103+CD11c+ DCs versus CD103−CD11c+. DCs is consistent with their well-known tolerogenic properties and their ability to induce Foxp3 in CD4+ T cells^32^. We thus measured Foxp3 expression in naive CD4+ T cells co-cultured with OVA_323–339_ peptide-loaded TCRγδ+LAP+ or TCRγδ+LAP− cells. We found that TCRγδ+LAP+ but not TCRγδ+LAP− cells induced Foxp3 expression in a fashion analogous to CD103+CD11c+ DCs (Fig. 4c). Because LAP has been reported to be important for Foxp3 induction in a cell-contact-dependent manner^33^, we investigated the requirement for LAP to induce Foxp3 by TCRγδ+LAP+ cells *in vitro*. Using a monoclonal anti-LAP antibody developed in our laboratory^34^, we found that the induction of Foxp3 in CD4+ T cells by TCRγδ+LAP+ cells was reduced by three-fold when LAP was blocked (Fig. 4d). To investigate whether the proliferative activity and Foxp3 induction by TCRγδ+LAP+ cells were dependent on MHC-II, we sorted TCRγδ+LAP+ cells from MHC-II−/− mice, pulsed them with OVA_323–339_ peptide, and co-cultured them with CellTrace Violet-labeled naive CD4+ T cells from OT-IIxFoxp3-GFP. We found that MHC-II+/+ but not MHC-II−/− TCRγδ+LAP+ cells pulsed with OVA_323–339_ peptide induced proliferation and Foxp3 expression in CD4 T cells (Fig. 4e,f). Thus, TCRγδ+LAP+ cells have MHC-II dependent APC properties.

To investigate whether TCRγδ+LAP+ cells could promote proliferation and Foxp3 induction in CD4 T cells *in vivo*, we co-transferred CellTrace Violet-labeled naive CD4+ T cells from OT-IIxFoxp3-GFP mice with either TCRγδ+LAP+ or TCRγδ+LAP− cells pulsed with OVA_323–339_ peptide to WT recipient mice and measured proliferation and Foxp3 expression in transferred CD4+ T cells 5 days later in the spleen. We found that both OVA_323–339_ peptide-pulsed TCRγδ+LAP+ and TCRγδ+LAP− cells induced CD4+ T cell proliferation *in vivo*, with greater proliferative activity induced by TCRγδ+LAP+ cells (Supplementary Fig. 9a). In addition, TCRγδ+LAP+ cells induced more Foxp3 than their LAP− counterparts *in vivo* (Supplementary Fig. 9b). Thus, LAP-expressing γδ T cells can function as APCs and induce CD4+Foxp3+ cells *in vivo*.

### TCRγδ+LAP+ cells arise from thymic γδT1 cells

**Figure 5:**
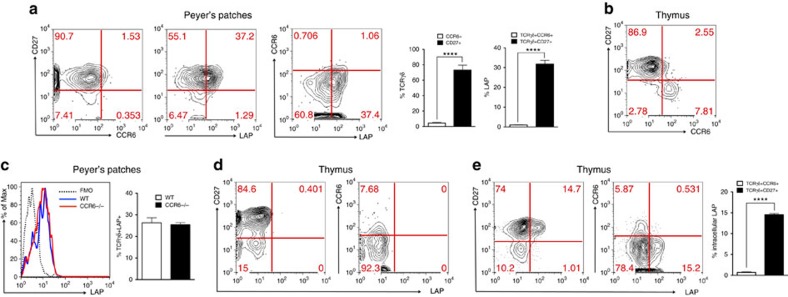
TCRγδ+LAP+ cells are originated from thymic γδT1 cells. (**a**) CD27 and CCR6 expression on γδ T cells (CD3+TCRγδ+) as well as expression of LAP on CD3+TCRγδ+CD27+ and CD3+TCRγδ+CCR6+ cells from PPs of naive C57BL/6 mice (*n*=9). (**b**) CD27 and CCR6 expression on thymic γδ T cells (CD3+TCRγδ+) from naive C57BL/6 mice (*n*=9). (**c**) LAP expression on γδ T cells (CD3+TCRγδ+) from WT and CCR6−/− mice (*n*=6 per group). (**d**) Surface LAP expression on thymic CD3+TCRγδ+ and CD3+TCRγδ+ cells from naive C57BL/6 mice (*n*=9). (**e**) Intracellular LAP expression in thymic CD3+TCRγδ+ and CD3+TCRγδ+ cells from naive C57BL/6 mice. Cells were first incubated with purified anti-LAP (clone TW7–16B4) to block surface LAP and then fixed/permeabilized and labeled with PE-anti-LAP antibody (*n*=6). These data are representative of at least three independent experiments. Data are shown as mean±s.e.m. Student’s *t*-test was used. ****P<0.0001.

**Figure 6:**
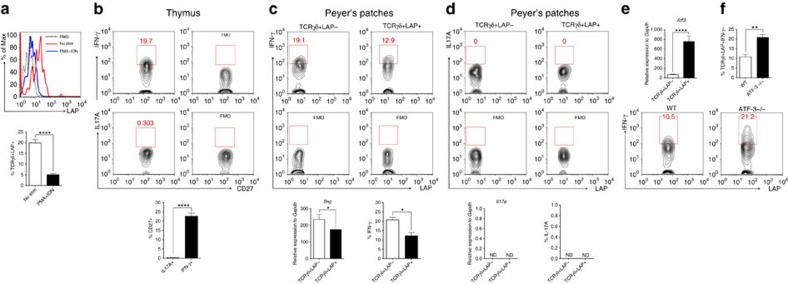
IFN-γ is downregulated in TCRγδ+LAP+ cells. (**a**) LAP expression on γδ T cells (CD3+TCRγδ+) from PPs with and without PMA+ION stimulation (*n*=6). (**b**–**d**) IFN-γ and IL17A expression (protein and quantitative RT–PCR) in non-stimulated CD3+TCRγδ+CD27+ cells from thymus (**b**) as well as CD3+TCRγδ+LAP− and CD3+TCRγδ+LAP+ cells from PPs (**c**,**d**; *n*=9; ND=non-detected). These data are representative of at least three independent experiments. (**e**) Quantitative RT–PCR analysis of *Atf3* mRNA from CD3+TCRγδ+LAP− and CD3+TCRγδ+LAP+ cells (*n*=pooled cells from 10 mice per experiment). (**f**) FACS plots and frequency of IFN-γ expression in non-stimulated CD3+TCRγδ+LAP+ cells from C57BL/6 WT or ATF-3−/− mice (*n*=9 per group). These data are representative of at least three independent experiments. Data are shown as mean±s.e.m. Student’s *t*-test was used. *P<0.05, **P<0.01, ****P<0.0001.

It has been shown that thymic γδ T cells can be divided into two subpopulations: γδT1 cells, characterized by the expression of CD27 and the production of IFN-γ (ref. 7); and γδT17 cells, which are CD27− ref. 7), express CCR6 and secrete IL-17 (ref. 6). Both subtypes are considered non-canonical γδ T cells and express Vγ1 and Vγ4 TCR chains^7^, which is consistent with what we observed in TCRγδ+LAP+ and TCRγδ+LAP− cells (Fig. 1g,h; Supplementary Fig. 4). To determine which subset gives rise to TCRγδ+LAP+ cells, we examined γδ T cells from PPs, the site where TCRγδ+LAP+ cells are in the greatest abundance. We found the majority of γδ T cells in PPs were positive for CD27, but negative for CCR6 (Fig. 5a). Thus, most of TCRγδ+LAP+ (as well as TCRγδ+LAP−) cells were γδT1 cells (Fig. 5a). Of note, 6–10% of thymic γδ T cells were γδT17 cells as they were negative for CD27 and expressed CCR6 (Fig. 5b). When we examined LAP expression on γδ T cells from CCR6−/− mice, there was no difference compared with WT mice (Fig. 5c), suggesting that TCRγδ+LAP+ cells arise from thymic γδT1 cells. The expression of surface LAP on γδ T cells most likely occurs in the periphery because neither thymic γδT1 nor γδT17 cells expressed LAP on the surface (Fig. 5d). Intracellular LAP expression was detected in 15% of thymic γδT1, but not in γδT17 cells (Fig. 5e), indicating that LAP is intrathymically induced but only expressed on the cell surface in the periphery. As previously reported, thymic γδT1 cells expressed IFN-γ, which increased after phorbol myristate acetate (PMA) and ionomycin (ION) stimulation, but virtually no IL17A (ref. 7). Because TCRγδ+LAP+ cells stimulated with PMA/ION downregulate LAP (Fig. 6a), we performed IFN-γ and IL17A intracellular staining from fresh *ex vivo* γδ T cells from both thymus and PPs. Thymic γδT1 cells produced IFN-γ, but not IL17A (Fig. 6b). TCRγδ+LAP+ cells expressed less IFN-γ, at both protein and mRNA levels than either thymic γδT1 or TCRγδ+LAP− cells (Fig. 6c), suggesting that LAP-expressing γδ T cells downregulate IFN-γ. IL17A protein and mRNA expression, however, was not observed in either TCRγδ+LAP+ or TCRγδ+LAP− γδ T cells (Fig. 6d). Taken together, these data indicate that thymic γδT1 cells acquire surface LAP in the periphery where IFN-γ is downregulated.

### TCRγδ+LAP+ cells downregulate IFN-γ through ATF-3

Our RNA-Seq data demonstrated that activating transcription factor 3 (ATF-3) was upregulated in TCRγδ+LAP+ cells (Table 1; Supplementary Data 1 and Supplementary Data 2). We focused on ATF-3 because it relates directly to a potential mechanism by which IFN-γ is downregulated in TCRγδ+LAP+ cells. ATF-3 is a member of the ATF/CREB family of basic leucine zipper transcription factors that has been shown to negatively modulate IFN-γ either indirectly by reducing cytokine production, including IL-12 (ref. 35), or directly by interacting with a cis-regulatory element of the IFN-γ gene^36^. We confirmed increased expression of ATF3 in TCRγδ+LAP+ cells by RT-PCR (Fig. 6e). Consistent with this, we found that PP from ATF-3−/− mice had twice as many IFN-γ-producing TCRγδ+LAP+ cells than WT mice (Fig. 6f), suggesting that ATF-3 plays an important role in down-modulating IFN-γ in TCRγδ+LAP+ cells.

### Thymic γδT1 cells are imprinted with gut-homing molecules

**Figure 7:**
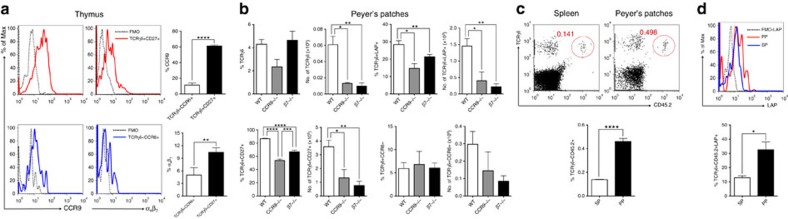
Expression of gut-homing molecules on γδ T cells. (**a**) CCR9 and α_4_β_7_ expression on thymic γδ T cells (CD3+TCRγδ+CD27+ and CD3+TCRγδ+CCR6+) from naive C57BL/6 mice (*n*=6). (**b**) Frequency and absolute number of total γδ T cells, CD3+TCRγδ+CD27+, CD3+TCRγδ+LAP+ and CD3+TCRγδ+CCR6+ cells from C57BL/6 WT, CCR9−/− and β_7_−/− mice in the PPs (*n*=5 per group). (**c**) FACS plot and frequency of transferred CD45.2+TCRγδ+CD27+ cells in the spleen and PPs of congenic CD45.1 mice 36 h after transfer (*n*=3). (**d**) Histogram and frequency of CD45.2+TCRγδ+LAP+ cells in the spleen and PPs of congenic CD45.1 mice 36 h after transfer (*n*=3). Data are shown as mean±s.e.m. One-way analysis of variance followed by Tukey multiple comparisons (**b**) and Student’s *t*-test (**a**,**c**,**d**) were used. *P<0.05, **P<0.01, ***P<0.001, ****P<0.0001.

Because the highest percentage of TCRγδ+LAP+ cells was found in PPs and SI-LP (Fig. 1a; Supplementary Fig. 1), we asked whether thymic γδ T cells expressed the CCL25 chemokine receptor CCR9 as well as the integrin α_4_β_7_, which are considered gut-homing molecules^37^. We found that expression of CCR9 and α_4_β_7_ was primarily detected on γδT1 cells with lower expression on γδT17 cells (Fig. 7a). To further investigate the role of CCR9 and α_4_β_7_ on homing of γδ T cells to the gut, CCR9−/− and β_7_−/− mice were used and we found reduced frequency/absolute number of γδT1 and TCRγδ+LAP+ cells in the PP (Fig. 7b). Because γδT1 cells correspond to the majority of γδ T cells in the PP (Fig. 5a; Fig. 6b), the absolute number of total γδ T cells was also decreased (Fig. 7b). In association with the smaller γδT1 and TCRγδ+LAP+ cell compartments in the gut of CCR9−/− and β_7_−/− mice, we found these γδ T cell populations increased in the spleen of both CCR9−/− and β_7_−/− mice (Supplementary Fig. 10a). Consistent with the fact that CCR9 and α_4_β_7_ are expressed less on γδT17 than γδT1 cells, neither frequency nor absolute number of γδT17 cells from CCR9−/− and β_7_−/− mice were altered in PPs, though number, but not percentage of these cells were increased in the spleen of CCR9−/− but not β_7_−/− mice (Fig. 7b; Supplementary Fig. 10a). To further confirm the gut-homing ability of γδT1 cells, we transferred sorted thymic TCRγδ+CD27+ cells from WT CD45.2 C57BL/6 mice to WT congenic CD45.1 mice. We then tracked the CD45.2+ cells 36 h later and found higher frequency of these cells in PPs (0.5%) than in spleen (0.15%; Fig. 7c) Moreover, transferred CD45.2+ γδ T cells found in PPs expressed more LAP than splenic CD45.2+ γδ T cells (Fig. 7d). Consistent with our observation that TCRγδ+LAP+ cells downregulate IFN-γ (Fig. 6b), transferred CD45.2+TCRγδ+LAP+ cells had significantly less IFN-γ than their LAP− counterpart (Supplementary Fig. 10b). Neither CD45.2+TCRγδ+LAP− nor CD45.2+TCRγδ+LAP+ cells expressed IL-17A (Supplementary Fig. 10c). PMA+ION was not used to stimulate these cells, since, as shown in Fig. 5f, LAP cannot be detected under these conditions. Thus, gut-homing γδT1 cells migrate to the periphery with preferential accumulation in the gut.

## Discussion

Gamma-delta (γδ) T cells are a unique subset of lymphocytes which originate in the thymus after recombination activating gene (RAG)-mediated V(D)J rearrangement^38^. γδ T cells are important in the immune response against pathogens and tumours^39^ and are enriched in the skin and mucosal tissues^40^. In addition to their cytotoxic characteristics, regulatory functions of γδ T cells have been described, although they are not completely understood^12^,^16^,^18^,^19^,^20^,^41^. Of note, we found increased expression of GzmA and B in TCRγδ+LAP+ cells suggesting that they may have cytotoxic properties, though this was not measured in our study. Foxp3 expression occurs in γδ T cells stimulated *in vitro* and a subset of IL-10-producing γδ T cells that protect mice liver from Listeria-elicited, CD8 T-mediated injury has been described^20^. Nonetheless, conflicting data have been reported in the literature regarding effector versus regulatory function of γδ T cells in models of disease in mice^8^,^10^,^20^,^42^. Here we describe a subset of regulatory γδ T cells in mice that are Foxp3 negative and express LAP. We also observed TCRγδ+LAP+ cells in human peripheral blood.

We found TCRγδ+LAP+ cells throughout the immune system with highest expression in PPs and SI-LP. Both sites play an important role in defense against pathogens and the induction of immunological tolerance^43^. Because γδ T cells have been shown to respond quickly to microbial and non-microbial tissue perturbation^39^, which is particularly important in highly antigen-exposed sites, TCRγδ+LAP+ cells may play a crucial role in gut homeostasis by providing a rapid regulatory response after encountering antigen. This is supported by the *in vivo* regulatory properties of TCRγδ+LAP+ cells in the CD4+CD45RBhigh cell transfer model of colitis^29^. In this model, TCRγδ+LAP+ cells decreased the inflammatory response caused by transferred CD4 T cells through reduction of pro-inflammatory cytokines such as IFN-γ, IL-17A, IL-6, TNF-α, CCL2 and CXCL10 and increase of the anti-inflammatory cytokines IL-10 and TGF-β1 mainly in the SI-LP. TCRγδ+LAP+ cells induced proliferation and Foxp3 expression in the transferred CD4 T cells in the SI-LP and spleen, but not in the LI-LP, suggesting that TCRγδ+LAP+ cells preferentially migrate to the SI-LP, where they control colitis by increasing the Foxp3+ Treg cell compartment and by switching the intestinal milieu from an inflammatory to a regulatory one. Splenic TCRγδ+LAP+ cells appear to play an important role in inducing CD4+Foxp3+ cells and controlling colitis because, although there is a lower frequency of TCRγδ+LAP+ cells in the spleen, the absolute number of TCRγδ+LAP+ cells is 5-fold more than in SI-LP. In the DSS model of colitis^30^, transfer of TCRγδ+LAP+ cells ameliorated disease. How TCRγδ+LAP+ cells exerted their regulatory activity in this model is not yet clear. In mice that were killed during the recovery phase of the colitis, we found differences in anti-inflammatory cytokines, such as IL-10 and TGF-β1 as well as IL-22, an important interleukin involved in the promotion of antimicrobial immunity, inflammation and tissue repair at barrier surfaces^31^. Of note, the mechanism by which DSS induces intestinal inflammation is believed to result from damage to the epithelial monolayer lining in the large intestine allowing the dissemination of pro-inflammatory intestinal contents (such as bacteria and their products) into underlying tissue^44^. Thus TCRγδ+LAP+ cells may control DSS-induced colitis by protecting gut epithelium. Furthermore, mice given TCRγδ+LAP+ cells had higher frequency and absolute cell number of CD4+Foxp3+ cells in the spleen, but no difference was seen in the LI-LP, the DSS target site. It is possible that analysis of CD4+Foxp3+ cells at earlier stages in the DSS-induced colitis model would show Treg cell expansion induced by TCRγδ+LAP+ cell treatment in the colonic lamina propria. Taken together, these data indicate that the regulatory effects induced by TCRγδ+LAP+ cells in DSS colitis is related to an increase of the Foxp3+ Treg cell compartment as well as production of anti-inflammatory and epithelium protective cytokines.

Suppressive activity of TCRγδ+LAP+ cells was not observed in a conventional *in vitro* suppression assay in which responder naïve CD4 T cells were stimulated with anti-CD3ε in the presence of APCs. Under these conditions TCRγδ+LAP+ cells induced proliferation of responder cells to a greater extent than TCRγδ+LAP− cells or controls. Furthermore, contrary to what we observed *in vivo*, under these *in vitro* conditions, Foxp3 expression was not induced in responder cells, suggesting that the *in vivo* regulatory function of TCRγδ+LAP+ cells involves more complex cell-cell interactions than *in vitro*. These data also suggest that TCRγδ+LAP+ cells acquire a pro-inflammatory phenotype following anti-CD3ε stimulation *in vitro*. Consistent with this, we found that TCRγδ+LAP+ cells stimulated *in vitro* with plate-bound anti-CD3ε/anti-CD28 produced less TGF-β1 and more TNF-α mRNA than freshly isolated TCRγδ+LAP+ cells. Accordingly, Foxp3 was induced when MOG specific CD4+ TCR Tg cells were cultured with freshly isolated TCRγδ+LAP+ cells. Thus, stimulation of TCRγδ+LAP+ cells with anti-CD3ε impairs their ability to induce CD4+Foxp3+ cells, but does not affect their ability to induce proliferation of CD4+ T cells. Foxp3 induction by TCRγδ+LAP+ cells was reversed by anti-LAP blocking antibody, indicating that induction of Foxp3 is mediated by LAP/TGF-β1, analogous to the infectious tolerance induced by CD4+Foxp3+ Treg cells which also relies on LAP/TGF-β1 expression^33^.

We found that TCRγδ+LAP+ cells upregulated antigen presentation-associated molecules including MHC-II, CD40 and CD86. Consistent with this, the APC-like function and Foxp3 induction capability of TCRγδ+LAP+ cells were lost when TCRγδ+LAP+ cells from MHC-II−/− mice were used. Of note, although TCRγδ+LAP− cells did not induce proliferation *in vitro*, they did induce proliferation *in vivo*. This difference may be related to the activation of TCRγδ+LAP− cells *in vivo*, which in turn would increase the basal expression of antigen presentation molecules and enhance their APC function^22^,^23^. However, because TCRγδ+LAP− cells do not express LAP, they do not have regulatory properties.

We found that thymic γδT1 (CD27+INF-γ+) cells gave rise to both TCRγδ+LAP+ and TCRγδ+LAP− cells. This is consistent with our observation that TCRγδ+LAP+ and TCRγδ+LAP− cells as well as γδT1 cells expressed Vγ1 and Vγ4 TCR chains, a characteristic of non-canonical γδ T cells^7^. Intracellular LAP was detected in 15% of thymic γδT1 cells and LAP was further upregulated after γδT1 cells migrated from the thymus to the periphery, primarily to the gut (PPs and SI-LP). Because GARP, a glycoprotein known to bind and attach LAP to the cell surface^27^ was not detected on thymic γδT1 cells, this may explain why thymic γδT1 cells do not express surface LAP. Thymic γδT1 cells expressed the gut-homing imprint molecules CCR9, the CCL25 chemokine receptor, and α_4_β_7_ integrin, which binds to the mucosal addressin cell adhesion molecule-1 (MAdCAM-1) expressed on the high endothelial venules of the PPs and gut lamina propria^45^. When γδT1 cells become TCRγδ+LAP+ cells, we observed downregulation of IFN-γ an effect that may be mediated by ATF-3. ATF-3 is an adaptive-response gene^46^ and may modulate IFN-γ production either indirectly by reducing the production of cytokines, such as IL-12 (ref. 35), or directly by targeting a cis-regulatory element in the IFN-γ gene (at least in NK cells)^36^. We found that ATF-3−/− mice had higher expression of IFN-γ in TCRγδ+LAP+ cells, suggesting that ATF-3 may have a direct effect on IFN-γ transcription in TCRγδ+LAP+ cells, as IL-12 is not required for IFN-γ expression in γδ T cells^7^,^47^,^48^.

Our data suggest that thymic γδT1 cells expressing CCR9 and α_4_β_7_ integrin migrate to the gut, upregulate GARP and LAP, downregulate IFN-γ via ATF-3 and acquire their APC properties. When TCRγδ+LAP+ cells present antigen to a CD4 T cell, TGF-β1 is released from LAP and induces Foxp3 in the CD4 T cell, rendering them regulatory. One of the major molecules that converts LAP to TGF-β1 is thrombospondin-1 (TSP-1)^49^,^50^,^51^. TSP-1 is expressed in both naive and activated CD4 T cells^52^,^53^ and activates TGF-β1 both *in vitro* and *in vivo*. Thus, the induction of Tregs by TCRγδ+LAP+ cells appears to be an important physiologic mechanism by which TCRγδ+LAP+ cells contribute to gut homeostasis.

In summary, our data identify TCRγδ+LAP+ cells as a new subset of γδ T cells with regulatory properties. The identification of TCRγδ+LAP+ cells provides a new avenue for understanding immune regulation and biologic processes linked to intestinal function and disease.

## Methods

### Mice

Male and female, 8–10-week-old and on a B6 genetic background mice were used in this study. C57BL/6 wild type, congenic CD45.1, RAG-1−/−, CCR6−/−, MHC-II−/− and β_7_−/− mice were purchased from the Jackson Laboratory. CCR9−/− mice were kindly provided by Dr Jesus Rivera-Nieves (University of California at San Diego—UCSD). ATF3−/−, Foxp3-GFP, OT-IIxFoxp3-GFP and 2D2xFoxp3-GFP mice were housed in a conventional specific pathogen-free facility at the Harvard Institutes of Medicine according to the animal protocol with the full knowledge and permission of the Standing Committee on Animals at Harvard Medical School.

### FACS and intracellular cytokine staining

A pool of cells from spleen and PPs or thymus of Foxp3-GFP and C57BL/6 mice was first enriched using CD4 microbeads (Foxp3-GFP) or TCRγδ isolation kit (C57BL/6; all from Miltenyi Biotec). Naive (CD4+CD62L+CD44-Foxp3− and CD4+Foxp3+ cells were sorted (FACS Aria II, BD Bioscience) with peridinin chlorophyll protein (PerCP)-conjugated anti-CD4 (RM4–5; 1:250), allophycocyanin (APC)-conjugated anti-CD62L (MEL-14; 1:250) and phycoerythrin (PE)-conjugated anti-CD44 (IM7; 1:500; all from BioLegend). CD4+Foxp3+ cells were sorted on the basis of GFP expression. TCRγδ+LAP+ and TCRγδ+LAP− T cells were sorted with Alexa Fluor 700 (AF700)-conjugated anti-CD3ε (eBio500A2; 1:100), APC-conjugated anti-TCRγδ (eBioGL3; 1:100) and PE-conjugated anti-latency-associated peptide (LAP)/TGF-β1 (TW7–16B4; 1:50; all from eBioscience). For TCRγδ cell sorting, dead cells were excluded on the basis of 7-AAD (1:25; BD Bioscience) staining. For intracellular cytokine staining, surface markers were stained for 25 min at 4 °C in Mg^2+^ and Ca^2+^ free HBSS with 2% FCS, 0.4% EDTA (0.5 M) and 2.5% HEPES (1 M) then were fixed in Cytoperm/Cytofix (eBioscience), permeabilized with Perm/Wash Buffer (eBiosciences) and stained with PE-Cy7-anti-IFN-γ (XMG1.2; 1:200) and FITC-anti-IL-17A (eBio17B7; 1:100; both from eBioscience) diluted in Perm/Wash buffer. In case of stimulation, the cells were stimulated for 4 h with PMA (phorbol 12-myristate 13-aceate; 50 ng ml^−1^; Sigma-Aldrich) and ionomycin (1 μM; Sigma-Aldrich) and a protein-transport inhibitor containing monensin (1 μg ml^−1^ GolgiStop; BD Biosciences) before detection by staining with antibodies. Flow-cytometric acquisition was performed on an LSRII (BD Bioscience) by using DIVA software (BD Bioscience) and data were analysed with FlowJo software versions 9.6.4 (TreeStar Inc). To show specificity of LAP staining in Fig. 1 and Supplementary Figs 1 and 2, cells were first incubated with anti-LAP mAb (TW7–16B4 for mouse; kindly provided by Dr Takatoku Oida; TW4-2F8 for human) for 20 min, washed and staining with surface markers, including either mouse Brilliant Violet 421 (BV421; TW7–16B4; 1:100) or human PE-anti-LAP (TW4-2F8; 1:100) antibodies. Other antibodies included: PerCP-anti-CD3ε (1452C11; 1:100), PE-anti-α_4_β_7_ (DATK32; 1:100), eFluor450-anti-CCR9 (CW-1.2; 1:100), PE-anti-GARP (YGIC86; 1:100), PE-anti-TCR Vγ2 (UC3–10A6; 1:100), FITC-anti-CD27 (LG.7F9; 1:100), PE-anti-CD27 (LG.7F9; 1:100), PE-Cy7-anti-CCR6 (R6H1; 1:100), APC-anti-CD45.2 (104; 1:100; all from eBioscience), Pacific Blue (PB)-anti-CD8a (53-6.7; 1:100), FITC-anti-TCRVγ1.1/Cr4 (2.11; 1:100), PE-Cy7-anti-I-A/I-E (M5/114.15.2; 1:200), PerCP-Cy5.5-anti-CD27 (LG.3A10; 1:100), PE-anti-CD103 (2E7; 1:100), FITC-anti-CD86 (GL-1; 1:100), FITC-anti-H-2Kb/H-2Db (28-8-6; 1:100; all from Biolegend), PE-anti-CD40 (3/23; 1:100; BD Bioscience).

### Human peripheral blood mononuclear cell LAP staining

We collected blood from healthy controls (age 25–35 years) upon informed consent. Peripheral blood mononuclear cells were obtained by Ficoll density gradient and cells were stained with eFluor450-anti-CD3 (OKT3; 1:100), FITC-anti-TCRγδ (B1.1; 1:100) and PE-anti-LAP (TW4-2F8; 1:100) for flow cytometric analysis.

### Purification and Cell transfer

Pooled cells from spleen and PPs or thymus of C57BL/6 mice were first enriched using CD4 microbeads and TCRγδ isolation kit (both from Miltenyi Biotec), as described above and then sorted. The purity of each population was >98% as analysed by flow cytometry. To evaluate the immunomodulatory effect of TCRγδ+LAP+ cells on DSS-induced colitis model, we transferred 1 × 10^5^ TCRγδ+LAP− or TCRγδ+LAP+ cells per animal intravenously. For the CD4+CD45RBhigh cell transfer-induced colitis model, we transferred 5 × 10^5^ CD4+CD45RBhigh cells per animal intraperitoneally and 2.5 × 10^5^ TCRγδ+LAP− or TCRγδ+LAP+ cells per animal intravenously. For CD45.2+TCRγδ+CD27+ cell transfer to CD45.1 congenic mice, 1 × 10^6^ cells per mouse intravenously were used. Intravenously and intraperitoneally, cell transfers were performed in 100 and 500 μl of phosphate-buffered saline (PBS), respectively.

### *In vitro* suppression assay

For suppression assays, sorted TCRγδ+LAP+, TCRγδ+LAP− or CD4+Foxp3+ cells were cultured at 1:1, 1:2, 1:4 and 1:8 ratio with syngeneic responder cells (CD4+CD62L+CD44-Foxp3−) previously stained with CellTrace Violet according to the manufacturers’ recommendation (CellTrace Violet proliferation kit, Invitrogen). Cells were stimulated with anti-CD3ε (1 μg ml^−1^; 145-2C11, BioLegend) in the presence of mitomycin-treated (50 μg ml^−1^) APCs in 200 μl of IMDM medium supplemented with 10% FBS in 96-well round-bottom plates. Proliferation and Foxp3 induction were assessed 72 h later by flow cytometry, based upon the dilution of the CellTrace violet dye.

In some experiments, naive cells from 2D2xFoxp3-GFP mice were sorted and co-cultured with sorted TCRγδ+LAP− or TCRγδ+LAP+ cells from WT mice in the absence of APCs and stimulated with myelin oligodendrocyte glycoprotein (MOG_35–55_ peptide, 20 μg ml^−1^) in 200 μl of IMDM medium supplemented with 10% FBS in 96-well round-bottom plates. Proliferation and Foxp3 expression were assessed 72 h later by flow cytometry, based upon the dilution of the CellTrace violet dye and GFP expression, respectively.

### *In vitro* activation of TCRγδ+LAP− and TCRγδ+LAP+ cells

Sorted TCRγδ+LAP− and TCRγδ+LAP+ cells were incubated for 3 days at 37 °C in the presence of plate-bound anti-CD3 and anti-CD28 (1 μg ml^−1^ each). On the fourth day, RNA was extracted as described below in the real-time PCR section.

### Uptake and presentation assays

CD103+CD11c+, CD103-CD11c+ dendritic cells and TCRγδ+LAP+, TCRγδ+LAP− cells were first enriched using CD11c microbeads or TCRγδ isolation kit (all from Miltenyi Biotec) and sorted. For uptake assay, TCRγδ+LAP− and TCRγδ+LAP+ cells were incubated for 3 h at 37 °C with 50 μg ml^−1^ of ovalbumin (OVA) coupled to Alexa Fluor 488 (Invitrogen) in a 96-well round-bottom plate. After incubation, cells were collected, thoroughly washed and analysed by flow cytometry. For presentation assay, sorted CD103-CD11c+, CD103+CD11c+ dendritic cells and TCRγδ+LAP+, TCRγδ+LAP− cells (from WT or MHC-II−/− mice) were first incubated overnight at 37 °C with 50 μg ml^−1^ of OVA_323–339_ peptide or medium only (unloaded cells as control) in a 96-well round-bottom plate. Next day, the cells were thoroughly washed and incubated at 1:1 ratio with sorted naive (CD4+CD62L+CD44-Foxp3−) cells from OT-IIxFoxp3-GFP mice previously stained with CellTrace Violet dye (Invitrogen) for 4 days. Proliferation and Foxp3 induction were then analysed by flow cytometry. In some experiments, purified anti-LAP mAb (TW7–16B4) was used to study the involvement of LAP in the Foxp3 induction by TCRγδ+LAP+ cells at a concentration of 30 μg ml^−1^.

### *In vivo* presentation and Foxp3 induction assays

For the *in vivo* presentation and Foxp3 induction study, sorted TCRγδ+LAP− or TCRγδ+LAP+ cells from C57BL/6 mice were first loaded overnight with 50 μg ml^−1^ of OVA_323–339_ peptide (Invivogen) or medium only (unloaded cells as control), thoroughly washed, and 1 × 10^5^ cells per animal were intravenously transferred together with 2 × 10^6^ CellTrace Violet (Invitrogen)-stained naive CD4 T cells (CD4+CD62L+CD44-Foxp3− from OT-IIxFoxp3-GFP mice) per animal in a volume of 100 μl. CellTrace violet staining was performed according to the manufacturers’ recommendation. The mice were killed 5 days later and the spleens removed for FACS analysis.

### Dextran sodium sulfate-induced colitis model

TCRγδ+LAP+ and TCRγδ+LAP− cells were sorted from C57BL/6 mice as described above and intravenously transferred to syngeneic mice at 5 × 10^4^ cells per animal in 100 μl of PBS at days 0 and 2 (Supplementary Fig. 6a). Colitis was induced by 3% (w/v) dextran sodium sulfate (DSS; molecular weight 36–50 kDa; MP Biomedicals, LLC) added to the drinking water for 7 consecutive days. Mice were weighted every day until the end of the experiment (14 days). At day 14, the mice were killed and the colons were removed for length measurement, histological analysis, RT–PCR and FACS.

### CD4+CD45RBhigh cell transfer-induced colitis model

CD4+CD25-CD45RBhigh cells were sorted from C57BL/6 mice using APC-anti-CD4 (GK1.5), PerCP-Cy5.5-anti-CD45RB (C363-16A) and FITC-anti-CD25 (PC61) and intraperitoneally transferred in syngeneic mice at 5 × 10^5^ cells per animal in 500 μl of PBS. Then TCRγδ+LAP+ and TCRγδ+LAP− cells also sorted from C57BL/6 mice were intravenously transferred to the mice at 5 × 10^4^ cells per animal in 100 μl of PBS at the day of CD4+CD25-CD45RBhigh cell transfer and once a week for the next 4 weeks (Fig. 2a). Mice weights were measured every week until the end of the experiment (7 weeks), when they were sacrificed and colons and small intestines removed for length measurement, histological analysis, RT-PCR and FACS as well as spleens removed for flow cytometric analyses.

### Histopathology

Colons and/or small intestines were excised from animals at the end of both colitis experiments, flushed with PBS, cut longitudinally, rolled into ‘Swiss rolls’ and immediately fixed in formaldehyde 4% for 48 h and kept in ethanol 70%. Samples were then embedded in paraffin and 5 μm were cut and stained with haematoxylin and eosin. Sections were evaluated for histopathological changes, such as tissue integrity and inflammatory cells infiltration after being loaded into an Aperio ScanScope XT (Aperio), scanned via the semi-automated method and checked for image quality using visual inspection.

### Real-time PCR

Naive CD4+CD62L+CD44-Foxp3−, CD4+Foxp3+, TCRγδ+LAP+ and TCRγδ+LAP− cells were sorted and RNA was extracted with a miRNeasy kit (Qiagen), then was reverse-transcribed with a high capacity cDNA reverse transcription kit (Applied Biosystems) and analysed by quantitative RT–PCR with a Vii 7 Real-time PCR system (Applied Biosystems) with the following primers and probes (from Applied Biosystems; identifier in parentheses): *Tgfb1* (Mm00441724_m1), *Ifng* (Mm00801778_m1), *Il17a* (Mm00439619_m1), *Atf3* (Mm00476032_m1), *Foxp3* (Mm00475156_m1), *Tnfa* (Mm004433258_m1), *Il6* (Mm00446191_m1), *Il10* (Mm00439616_m1), *Il22* (Mm00444241_m1), *Ccl2* (Mm00441242_m1), *Ccl5* (01302427_m1) and *Cxcl10* (Mm00445235_m1). The comparative threshold cycle method and the internal control *Gapdh* (Mm99999915-g1) was used for normalization of the target genes.

### Expression analysis of TCRγδ+LAP+ versus TCRγδ+LAP− cells using RNA-Seq

Total RNA samples were supplied to the Broad Institute's Genomics Platform and were QC’d by Agilent Bioanalyzer for RNA Integrity Scores (RIN>6), and normalized by Nanodrop to a minimum of 5 ng μl^-1^ and 250 ng. Libraries were constructed using Illumina’s TruSeq kit with Poly A selection, pooled and sequenced on the Illumina HiSeq 2000 with 76 bp paired-end reads to a read coverage of 15 M reads per sample.

After read preprocessing and GC bias removal, we processed our sequencing data using the latest Tuxedo RNA-Seq pipeline^54^, in particular, TopHat v2.0.11, Bowtie v2.2.2.0, and Cufflinks v2.2.1. We aligned our reads to mouse genome version GRCm38.p2 (mm10) with the Gencode GRCm38M2 gene set as annotation. Using Cuffdiff's traditional FPKM with a pooled replicate model, we generated signatures of differentially expressed genes for TCRγδ+LAP+ versus TCRγδ+LAP− cells. In addition to Cuffdiff’s significance measure, we chose to disregard low expression genes that did not have an arbitrary minimum absolute difference of 1 FPKM between expression values as cutoff to account for detection noise. Using these criteria, we identified a signature of 41 genes that were enriched specifically in TCRγδ+LAP+ cell samples with *q*<0.05, while 407 genes were enriched in TCRγδ+LAP+ cells with *P*<0.05. We used the likelihood function proposed by Trapnell *et al.*^55^ and Roberts *et al.*^56^ to calculate *P* and *q* values. To remove ‘infinity’ values of log2-fold change for plotting, we added a constant of 0.001 to all the expression values and then recalculated the log2-fold-expression difference.

### Statistics

GraphPad Prism 6.0 was used for statistical analysis (unpaired, two-tailed Student’s *t*-test or one-way analysis of variance, followed by Tukey multiple comparisons). For weight loss experiments, two-way analysis of variance was used. Differences were considered statistically significant with a *P* value of less than 0.05.

## Additional information

**How to cite this article:** Rezende, R. M. *et al.* Identification and characterization of latency-associated peptide-expressing γδ T cells. *Nat. Commun.* 6:8726 doi: 10.1038/ncomms9726 (2015).

## Supplementary Material

Supplementary Figures Supplementary Figures 1-10

Supplementary Data 1 Differential gene expression analysis (RNA-Seq) of TCRγδ+LAP- and TCRγδ+LAP+ cells.

Supplementary Data 2 Gene signature enriched in TCRγδ+LAP+ cells with q<0.05.

## Figures and Tables

**Table 1:** Transcriptional signatures of TCRγδ+LAP− and TCRγδ+LAP+ cells.

**Gene**	**LAP−**	**LAP+**	**LAP+/LAP− log2 (fold change)**
Itgae	57.640	150.950	1.389
Atf3	9.123	29.458	1.691
Cd81	5.272	17.706	1.748
Cd86	2.133	7.197	1.754
Cd244	9.620	33.830	1.815
Lag3	6.360	30.390	2.257
H2-Eb2	0.700	3.570	2.350
Cd83	2.939	15.285	2.379
H2-DMb1	1.143	7.794	2.769
H2-DMb2	1.398	9.625	2.783
Cd74	32.320	293.340	3.182
H2-Eb1	8.276	78.555	3.247
H2-Ab1	8.336	80.864	3.278
H2-Aa	10.520	107.540	3.354
Cd40	0.190	2.18	3.540
Apoe	8.640	108.030	3.645
Gzmb	25.390	336.200	3.727
Gzma	100.620	1,416.50	3.815

RNA-Seq expression analyses of upregulated genes in TCRγδ+LAP− and TCRγδ+LAP+ cells with *P*<0.05.
