# Supplementary material for: Identification and characterization of latency-associated peptide-expressing γδ T cells
Source: Nat Commun. 2015 Dec 8;6:8726. doi: 10.1038/ncomms9726 (PMC4686827; doi:10.1038/ncomms9726)
Supplement: Supplementary Figures — 1-10 [file ncomms9726-s1.pdf]

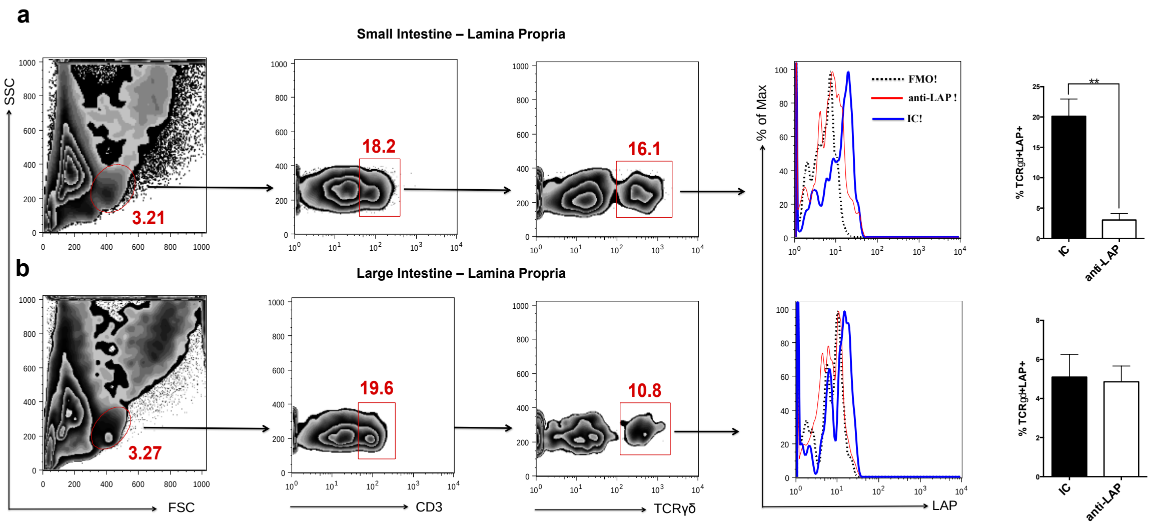

Supplementary Figure 1 LAP expression on γδ T cells from small intestine and large intestine lamina propria. **(a, b)** SI-LP **(a)** and LI-LP **(b)** showing the scheme for LAP gating. Anti-LAP blocking antibody (or isotype control (IC)) was used to show the specificity of LAP staining (n=5). Data are shown as mean $\pm$ SEM and are representative of at least 3 independent experiments. Student's *t*-test was used. ** $p<0.01$.

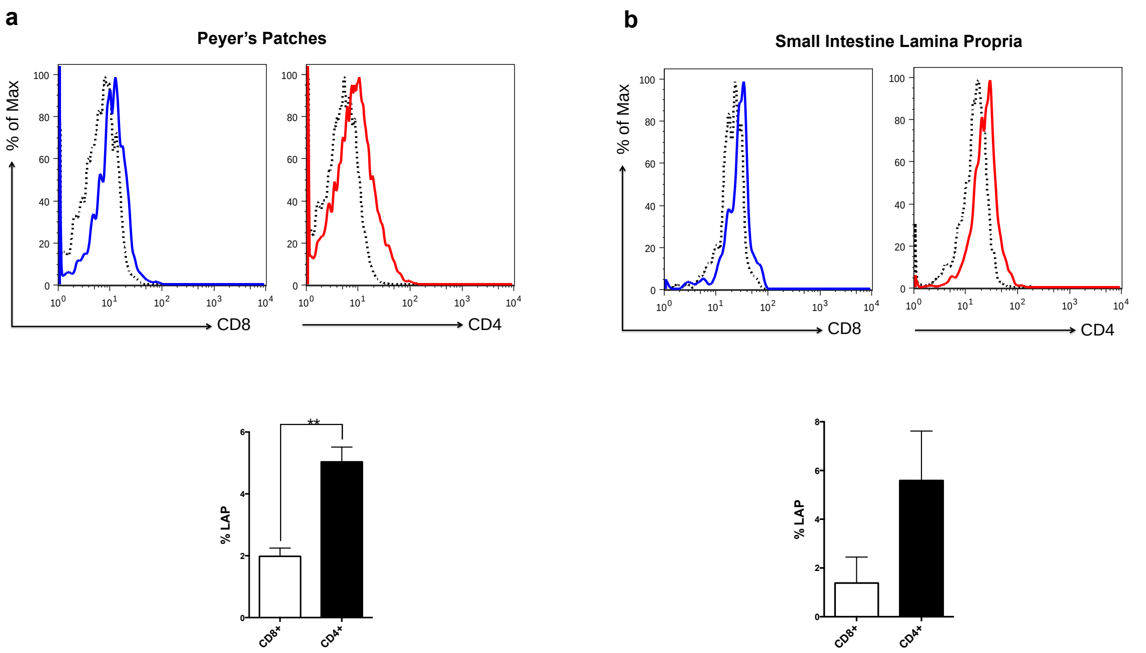

Supplementary Figure 2 LAP is only marginally expressed on CD4 and CD8 T cells in the Peyer's patches and small intestine lamina propria. **(a, b)** Histograms and frequency of LAP expression on CD4 and CD8 T cells in PP **(a)** and SI-LP **(b)** (n=5). Data are shown as mean ± SEM and are representative of at least 3 independent experiments. Student's *t*-test was used. ** p<0.01.

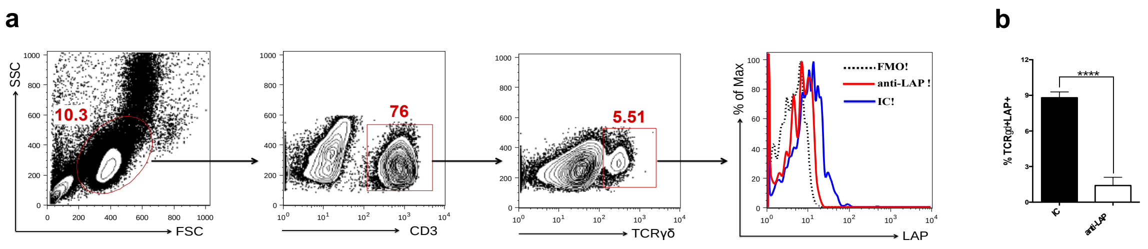

Supplementary Figure 3 LAP is expressed on human peripheral blood γδ T cells. **(a)** Human peripheral blood showing the scheme for LAP gating. Anti-LAP blocking antibody (or isotype control (IC)) was used to show the specificity of LAP staining. **(b)** LAP expression on human peripheral blood γδ T cells (n=5). Data are shown as mean ± SEM and are representative of at least 3 independent experiments. Student's *t*-test **(b)** was used. **** $p<0.0001$.

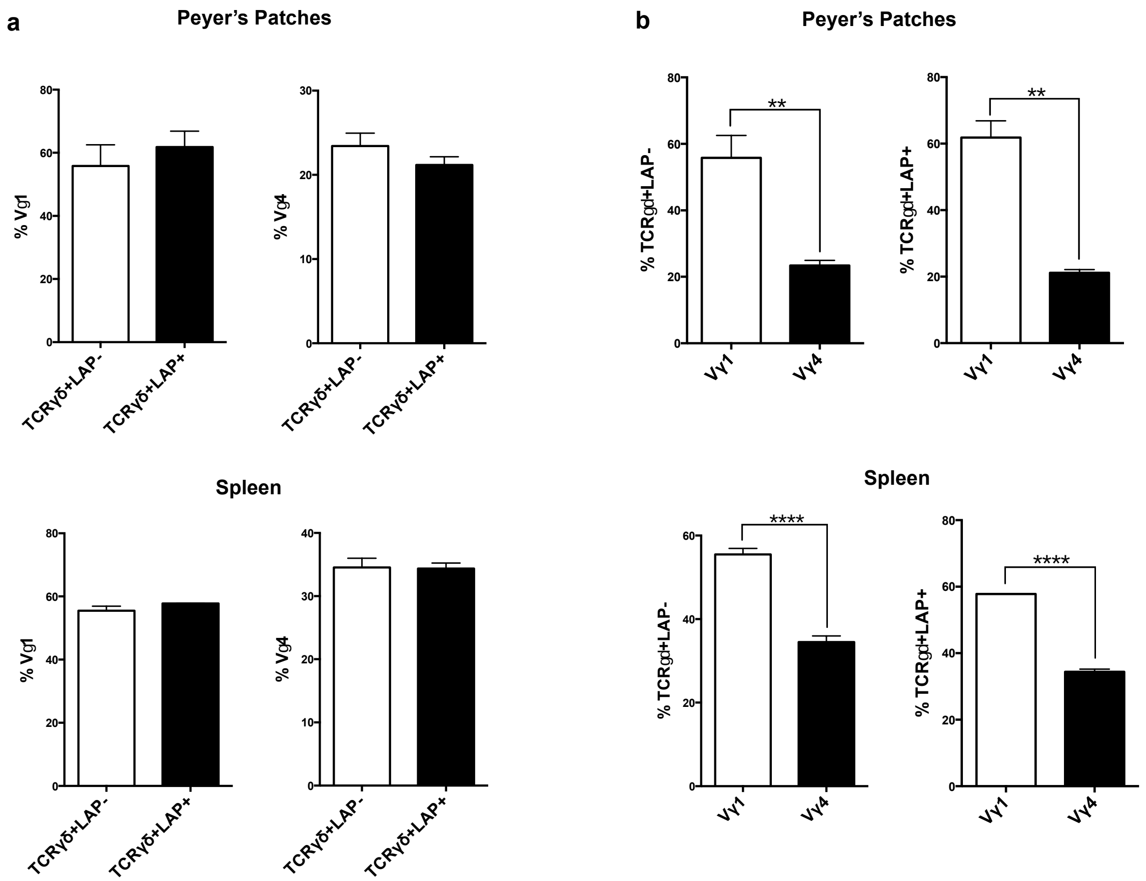

Supplementary Figure 4 Vγ1 and Vγ4 TCR chains expression on LAP- and TCRγδ+LAP+ cells. **(a)** Frequency of Vγ1 and Vγ4 TCR chains expression on TCRγδ+LAP- and TCRγδ+LAP+ cells from Peyer's patches **(top)** and spleen **(bottom)** of C57BL/6 WT mice. **(b)** Comparison between frequency of Vγ1 and Vγ4 TCR chains expression on TCRγδ+LAP- and TCRγδ+LAP+ cells from Peyer's patches **(top)** and spleen **(bottom)** of C57BL/6 WT mice. Data are shown as mean ± SEM and are representative of at least 2 independent experiments. Student's *t*-test was used. ** $p<0.01$, **** $p<0.0001$.

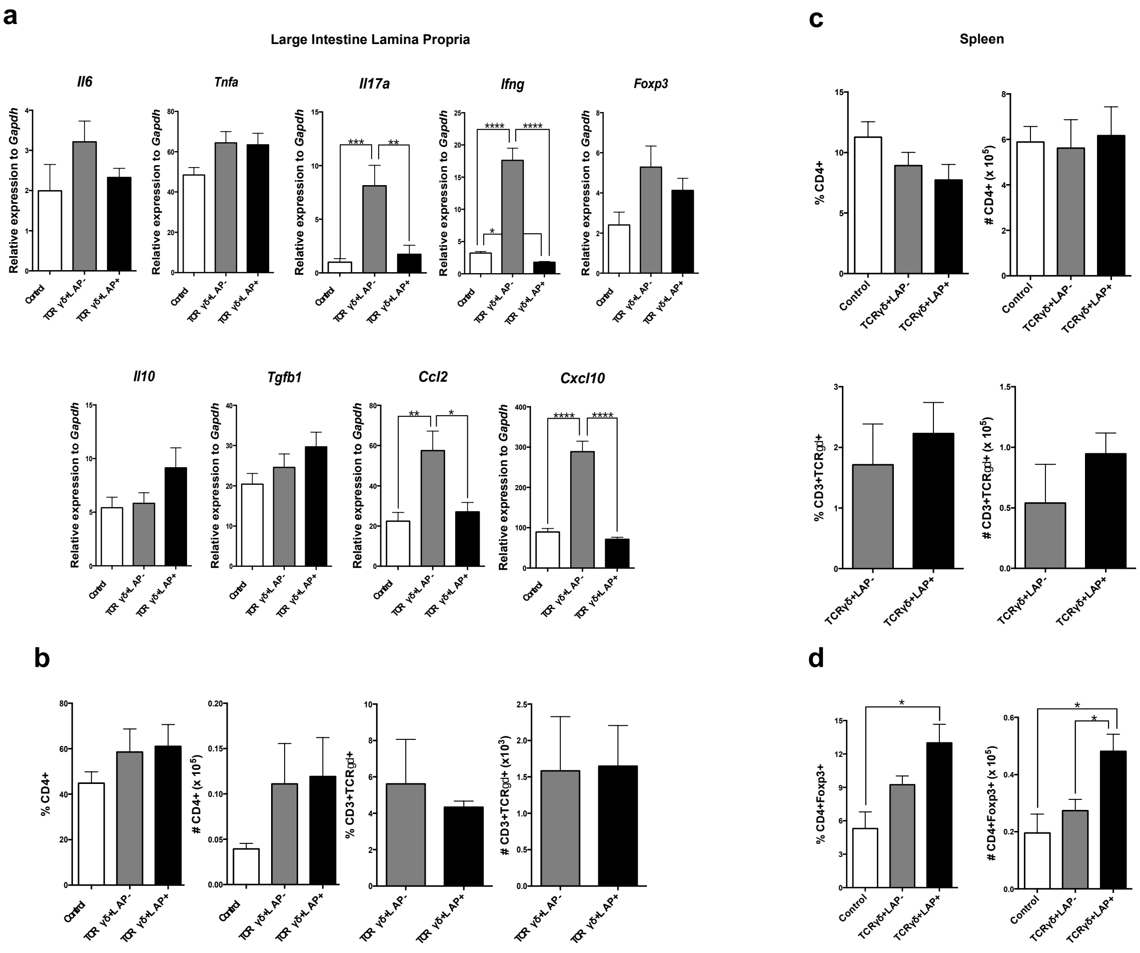

Supplementary Figure 5 Inflammatory profile induced by TCRγδ+LAP+ cells in the large intestine lamina propria and spleen of cell transfer-induced colitis mice. **(a)** Quantitative RT-PCR analysis of pro-inflammatory and anti-inflammatory cytokines from LI-LP. **(b, c)** Frequency and absolute number of total transferred CD4 T cells as well as total transferred CD3+TCRγδ+ cells in LI-LP **(b)** and spleen **(c)**. **(d)** Frequency and absolute number of CD4+Foxp3+ cells in spleen of cell transfer-induced colitis mice. Data are shown as mean ± SEM (n=4 for naïve; n=5 for control and TCRγδ+LAP- groups; n=3 for TCRγδ+LAP+ group) and are representative of 3 independent experiments. One-way ANOVA followed by Tukey multiple comparisons **(a; b left panels; c top panels; d)** and Student's *t*-test **(b right panels; c bottom panels)** were used. * $p<0.05$, ** $p<0.01$, *** $p<0.001$, **** $p<0.0001$.

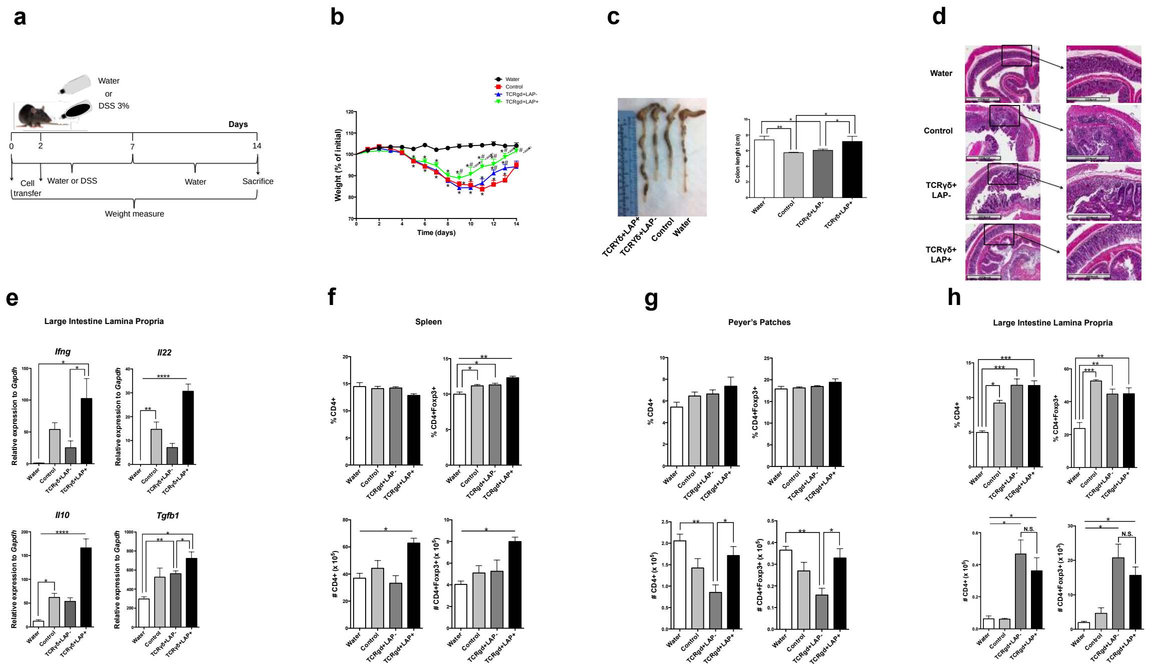

Supplementary Figure 6 TCRγδ+LAP+ cells ameliorate DSS-induced colitis. **(a)** Schematic protocol of DSS-induced colitis and γδ T cells adoptive transfer. **(b)** Body weight (% of initial weight) was measured throughout the experiment. Graph shows the mean $\pm$ SEM of water-treated (Water), DSS-treated only (Control), DSS-treated and CD3+TCRγδ+LAP- cells transferred (TCRγδ+LAP-) and DSS-treated and CD3+TCRγδ+LAP+ cells transferred (TCRγδ+LAP+) groups. **(c, d)** At day 14, mice were sacrificed and large intestines removed for length measurement **(c)** and hematoxylin-eosin staining (**d**; serial sections of 5µm; magnification of 40X (left panels, scale bar=600 µm) and 100X (right panels, scale bar=300 µm)). **(e)** Quantitative RT-PCR analysis of pro-inflammatory and anti-inflammatory cytokine mRNAs from LI-LP at day 14. **(f)** Frequency and absolute number of total CD4 T cells and CD4+Foxp3+ cells in spleen of DSS-induced colitis mice. Data are shown as mean $\pm$ SEM (n=9 for water; n=15 for control and TCRγδ+LAP- groups; n=9 for TCRγδ+LAP+ group) and are representative of 3 independent experiments. Two-way ANOVA **(b)** and One-way ANOVA followed by Tukey multiple comparisons **(c, e-f)** were used. * Statistically different from naïve group; # statistically different from control group ($p<0.05$); • statistically different from TCRγδ+LAP- group ($p<0.05$). * $p<0.05$, ** $p<0.01$, *** $p<0.001$, **** $p<0.0001$.

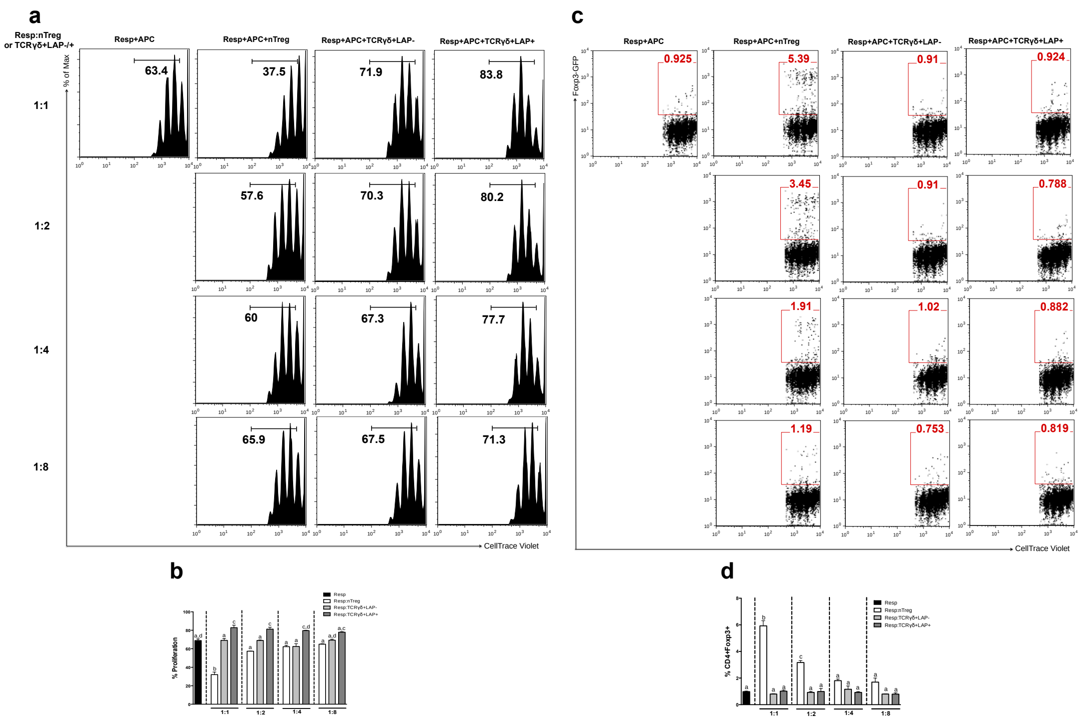

Supplementary Figure 7 TCRγδ+LAP+ cells induce proliferation of CD4 T cells *in vitro*. **(a, b)** FACS plots **(a)** and frequency **(b)** of CellTrace Violet-stained naive CD4 T cells (from Foxp3-GFP mice) proliferation after 3 days of co-culture with CD3+TCRγδ+LAP-, CD3+TCRγδ+LAP+ (from C57BL/6 WT mice) or CD4+Foxp3+ Treg cells (from Foxp3-GFP mice) in the presence of mitomycin-treated APCs at 1:1, 1:2, 1:4 and 1:8 ratios and stimulated with 1 μg/ml of purified anti-CD3ε. **(c, d)** FACS plots **(c)** and frequency **(d)** of Foxp3 induction in CellTrace Violet-stained naive CD4 T cells (from Foxp3-GFP mice) after 3 days of co-culture with CD3+TCRγδ+LAP-, CD3+TCRγδ+LAP+ (from C57BL/6 WT mice) or CD4+Foxp3+ Treg cells (from Foxp3-GFP mice) in the presence of mitomycin-treated APCs at 1:1, 1:2, 1:4 and 1:8 ratios and stimulated with 1 μg/ml of purified anti-CD3ε (n=pooled cells from 10 mice/experiment). Data are shown as mean ± SEM and are representative of at least 4 independent experiments. One-way ANOVA followed by Tukey multiple comparisons **(b, d)**. Bars marked with same letter are not statistically different from each other. Bars marked with different letters are statistically different from each other, $p<0.05$.

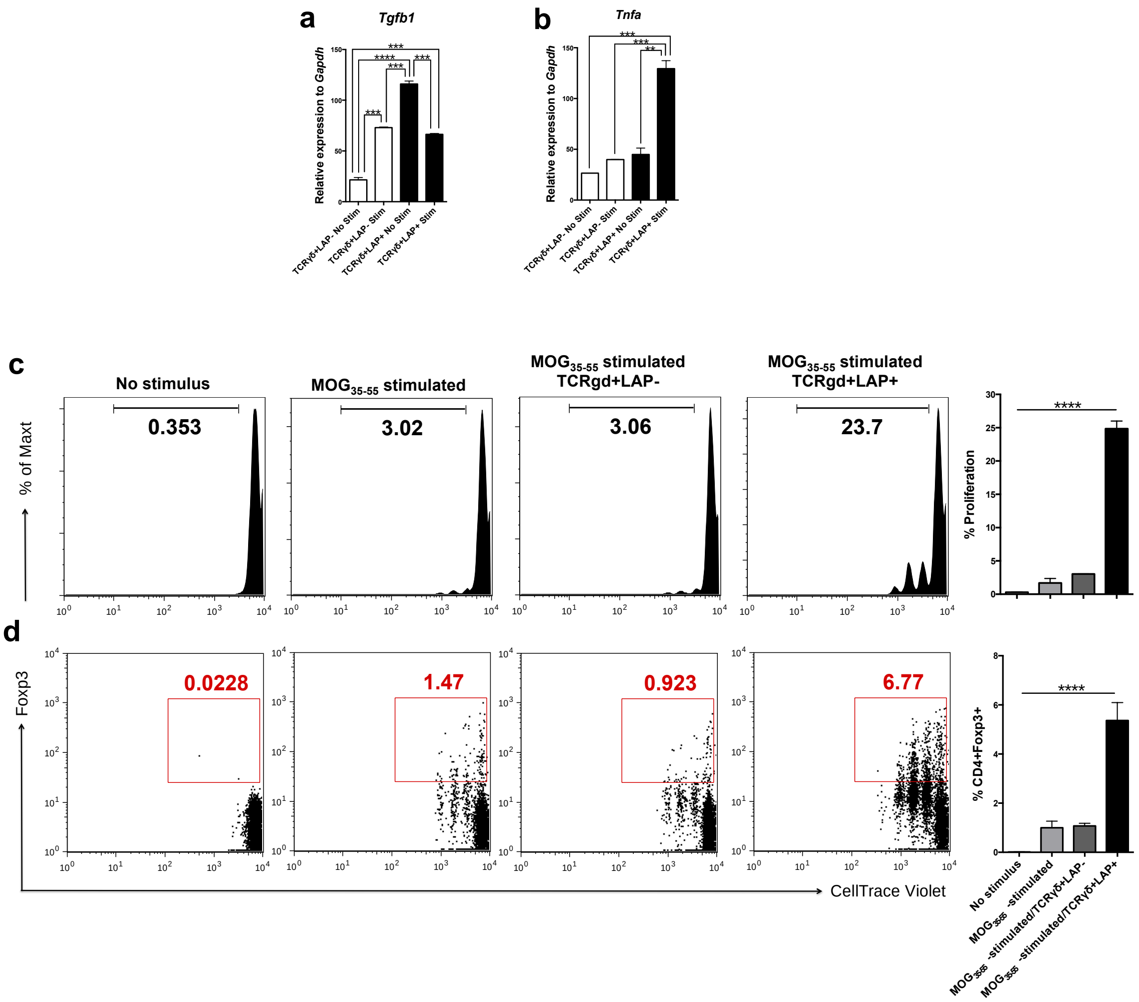

Supplementary Figure 8 *In vitro* activation renders TCRγδ+LAP+ cells pro-inflammatory. **(a, b)** Quantitative RT-PCR analysis of *Tgfb1* **(a)** and *Tnfa* **(b)** mRNAs from TCRγδ+LAP- and TCRγδ+LAP+ cells activated *in vitro* with plate-bound anti-CD3ε and anti-CD28 (1 μg/ml each) for 3 days (n=pooled cells from 10 mice/experiment). **(c, d)** Histograms and FACS plots of proliferation **(c)** and Foxp3 induction **(d)** by TCRγδ+LAP+, but not by TCRγδ+LAP- cells from WT C57BL/6 mice in CellTrace Violet-stained CD4 T cells from 2D2xFoxp3-GFP mice co-cultured in the absence of APC and stimulated with 20 μg/ml of MOG_{35-55} peptide (n=pooled cells from 10 mice/experiment). Data are shown as mean ± SEM and are representative of 2 independent experiments. One-way ANOVA followed by Tukey multiple comparisons. ** $p<0.01$, *** $p<0.001$, **** $p<0.0001$.

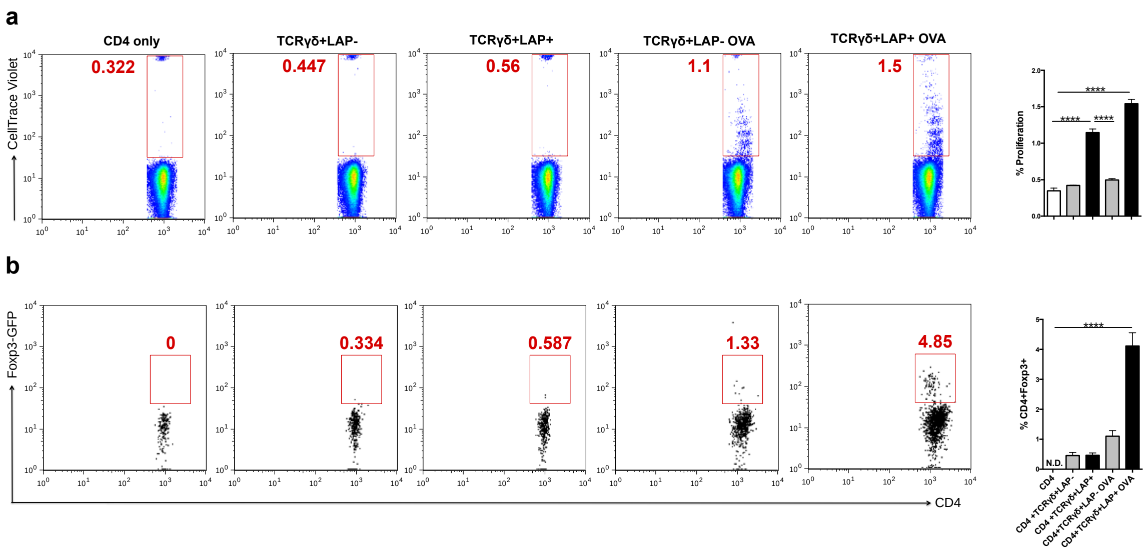

Supplementary Figure 9 TCRγδ+LAP+ cells induce proliferation and Foxp3 expression in CD4 T cell *in vivo*. **(a, b)** *In vivo* proliferation **(a)** and Foxp3 expression **(b)** in CellTrace Violet-stained naïve CD4 T cells from OT-IIxFoxp3-GFP mice co-transferred with $OVA_{323-339}$-loaded (or not) CD3+TCRγδ+LAP- or CD3+TCRγδ+LAP+ cells from C57BL/6 WT mice. Spleens were removed 5 days thereafter for flow cytometry analysis (n=pooled cells from 10 mice/experiment). Data are shown as mean $\pm$ SEM and are representative of at least 3 independent experiments. One-way ANOVA followed by Tukey multiple comparisons. **** $p<0.0001$.

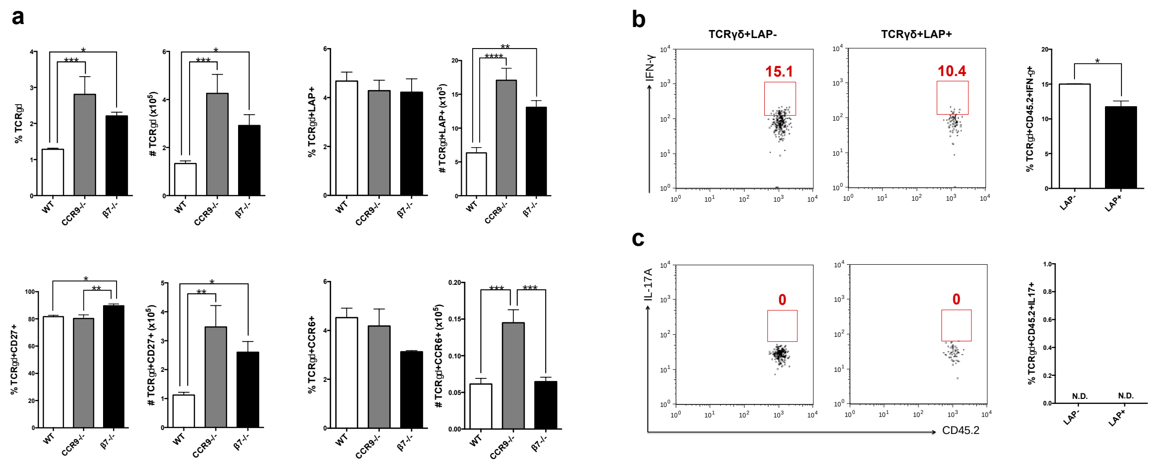

Supplementary Figure 10 Migratory characteristics of γδ T cells **(a)** Frequency and absolute number of total γδ T cells, CD3+TCRγδ+CD27+, CD3+TCRγδ+LAP+ and CD3+TCRγδ+CCR6+ cells from C57BL/6 WT, CCR9-/- and β_7-/- mice in the spleen (n=5/group). **(b, c)** FACS plot and frequency of transferred un-stimulated CD45.2+TCRγδ+IFN-γ+ **(b)** and CD45.2+TCRγδ+IL-17A cells in the PP **(c)** of congenic CD45.1 mice 36h after transfer (n=3). Data are shown as mean ± SEM. One-way ANOVA followed by Tukey multiple comparisons **(a)** and Student's *t*-test **(b-c)** were used. * p<0.05, ** p<0.01, *** p<0.001, **** p<0.0001.
